# Design of a Piezoelectric Accelerometer with High Sensitivity and Low Transverse Effect

**DOI:** 10.3390/s16101587

**Published:** 2016-09-26

**Authors:** Bian Tian, Hanyue Liu, Ning Yang, Yulong Zhao, Zhuangde Jiang

**Affiliations:** State Key Laboratory for Mechanical Manufacturing Systems Egineering, Xi’an Jiaotong University, Xi’an 710049, China; t.b12@mail.xjtu.edu.cn (B.T.); yn5653902@stu.xjtu.edu.cn (N.Y.); zhaoyulong@mail.xjtu.edu.cn (Y.Z.); zdjiang@mail.xjtu.edu.cn (Z.J.)

**Keywords:** piezoelectric accelerometer, high sensitivity, low resonance frequency, low transverse effect

## Abstract

In order to meet the requirements of cable fault detection, a new structure of piezoelectric accelerometer was designed and analyzed in detail. The structure was composed of a seismic mass, two sensitive beams, and two added beams. Then, simulations including the maximum stress, natural frequency, and output voltage were carried out. Moreover, comparisons with traditional structures of piezoelectric accelerometer were made. To verify which vibration mode is the dominant one on the acceleration and the space between the mass and glass, mode analysis and deflection analysis were carried out. Fabricated on an n-type single crystal silicon wafer, the sensor chips were wire-bonged to printed circuit boards (PCBs) and simply packaged for experiments. Finally, a vibration test was conducted. The results show that the proposed piezoelectric accelerometer has high sensitivity, low resonance frequency, and low transverse effect.

## 1. Introduction

With the development of urbanization, cables have gradually been replacing overhead lines due to their merits of being a reliable power supply, having a simple operation, and being a less occupying area. However, cable faults occur frequently because of the manufacturing processes, the operation environment, and insulation aging problems [1]. Moreover, cables are usually buried in the ground. Once the faults occur, they cannot be directly found by observation. Thus, methods to find faults quickly and accurately has become significantly important.

When cable faults occur, the electrical insulating layer breaks down, which causes an electric spark and generates a weak vibration [2]. The weak vibration caused by an electric spark can be measured as a characteristic signal, which can be extracted by vibration sensors or accelerometers [3]. Normally, the weak vibration signal is minute, directional, and low frequency (300~500 Hz) [4]. Therefore, the sensors should have the characteristics of high sensitivity, low transverse effect, and low resonance frequency response. In order to meet the requirements of the working environment, a new acceleration sensor was designed to detect a weak vibration signal.

Piezoelectric thin films have caused great interest in the design of accelerometers due to their potentially high sensitivity [5]. Several groups have previously reported on the use of piezoelectric thin films accelerometers. Eichner et al. [6] designed and fabricated bulk-micro machined accelerometers. A seismic mass and two silicon beams were used as the sensing structure; an average sensitivity of 0.1 mV/g was measured. Yu et al. [7] presented and fabricated a PZT (piezoelectric lead zirconate titanate) microelectromechanical accelerometer using interdigitated electrodes, which resulted in high acceleration sensitivity. The voltage sensitivities in the range of 1.3–7.86 mV/g with corresponding resonance frequencies in the range of 23–12 kHz were obtained. A bimorph tri-axis piezoelectric accelerometer with high sensitivity, low cross-axial sensitivity, and compact size was fabricated by Zou et al. [8]. The un-amplified sensitivities of the *X*-, *Y*-, and *Z*-axes electrodes in response to accelerations in *X*-, *Y*-, and *Z*-axes were 0.93 mV/g, 1.13 mV/g and 0.88 mV/g, respectively. Moreover, the cross-axis sensitivity was less than 15%. In this study, a novel structure for piezoelectric accelerometer on the basis of piezoelectric effect of piezoelectric material was established to further improve the sensitivity and reduce the transverse effect, including the principle of the piezoelectric accelerometer. Then, according to the FEM (finite element method), stress status, resonance frequency, and output voltage of different piezoelectric accelerometers were simulated and analyzed in detail. In accordance with the analysis results, the optimal structure of the piezoelectric accelerometer was obtained.

## 2. Design

The piezoelectric accelerometer is generally composed of a seismic mass, one or several beams, and piezoelectric thin films. When an acceleration signal is applied on the seismic mass, the force generated by the mass causes the beams to bend. Then, the piezoelectric thin films are under strain. Through the piezoelectric effect, the strain in the piezoelectric thin films is converted to an electrical charge [9]. By detecting the output voltage, the acceleration is obtained.

Commonly, an ideal acceleration sensor is only sensitive to the vibration signal that is perpendicular to the plane of the device, and it is not sensitive to the vibration signal in other directions. In fact, most acceleration sensors developed in the past could not avoid the transverse effect. The reason for accelerometer with transverse effect is mainly due to the fact that the center of the seismic mass is not in the same plane as that of the beams. When transverse acceleration is applied to the accelerometer, the seismic mass rotates around one axis, producing transverse interference. The transverse effect is described by the transverse effect coefficient [10,11]. The transverse effect coefficient is defined as

(1) $$R_{\mathrm{ST}}=\frac{\varepsilon \prime }{\varepsilon }.$$

Here, $R_{\mathrm{ST}}$ is the transverse effect coefficient, ε′ is the transverse strain, and ε is the longitudinal strain. In this paper, the longitudinal strain direction is the *Z* direction. The *X* direction and the *Y* direction are the two transverse strain directions. The transverse effect coefficient describes the influence of the transverse acceleration on the output of the sensors. The smaller the value is, the better the accelerometer performs.

A traditional accelerometer consists of a single sensitive beam, double sensitive beams, or four sensitive beams. The piezoelectric accelerometer with a single sensitive beam has a narrow bandwidth. Thus, the measuring range is narrow. The sensitivity of the piezoelectric accelerometer with double sensitive beams is not so high, but the transverse effect coefficient is quite large, which is susceptible to the lateral acceleration. The transverse coefficient of the piezoelectric accelerometer with four sensitive beams is small, but the sensitivity is low, as compared to a single sensitive beam and double sensitive beams [4].

**Figure 1:**
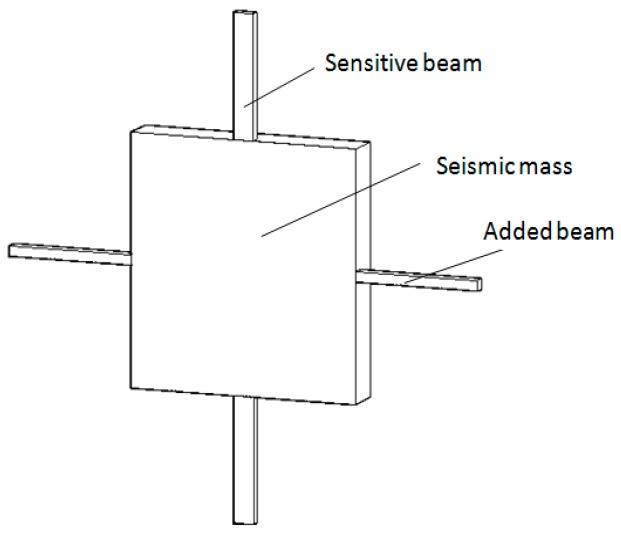
Diagram of the design structure.

To overcome defects of these structures, a structure with two sensitive beams and two added beams were designed, as shown in Figure 1. The seismic mass is suspended by the two sensitive beams and the two added beams. The length of the sensitive beams is shorter than that of the added beams while the width is larger. In addition, the thickness of both beams is the same.

**Figure 2:**
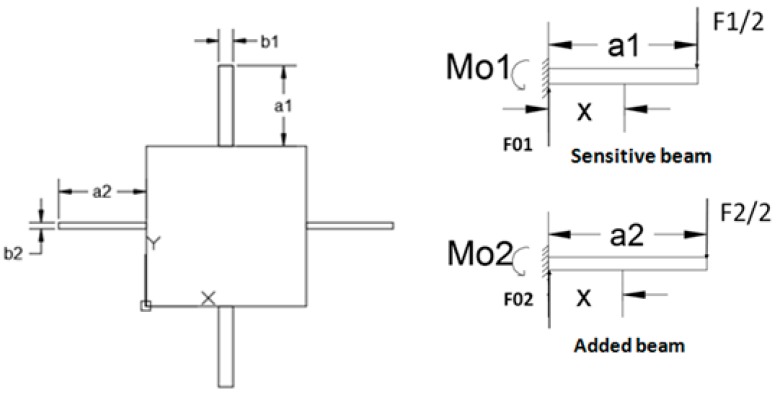
Mechanical analysis of the structure.

When the acceleration along *Z*-axis (the normal direction to the sensor chip) is applied on the chip, the beams is involved in a bending movement along with the vertical of the seismic mass. Since there is no rotation in the movement of the seismic mass, displacements of the beams are the same. Dimensions (length × width × thickness) of the sensitive beams and the added beams are a_1_ × b_1_ × h and a_2_ × b_2_ × h, respectively. Let F_1_ and F_2_ be the total forces applied to the sensitive beams and the added beams, as shown in Figure 2. In the analytical model, the mass of the beams and bending of the seismic mass are neglected. According to the basic principle of mechanics and under the assumption of small deflection [12], the following equations can be derived:

(2) $${\mathrm{EI}}_{1}\omega_{1}^{\prime \prime }\left(x\right)\;=\;\frac{1}{2}F_{1}x\;-M_{o1}\left(0\;\leq \;x\;\leq \;a_{1}\right),$$

(3) $${\mathrm{EI}}_{1}\omega_{2}^{\prime \prime }\left(y\right)\;=\;\frac{1}{2}F_{1}y\;-{\;M}_{o2}\left(0\;\leq \;y\;\leq \;a_{2}\right),$$

(4) $$F_{1}+F_{2}=\;F\;=\;\mathrm{ma},\;\mathrm{and}$$

(5) $$\omega_{1}\left(a_{1}\right)={\;\omega }_{2}\left(a_{2}\right),$$

where w_1_(x) and w_2_(y) are the displacement of the sensitive beams and the added beams, E is Young’s modulus of Si, M_01_ and M_02_ are restrictive moments to be determined, I_i_ = b_i_h^3^/12(i = 1,2). The boundary conditions for these equations are

(6) $$\omega_{1}\left(0\right)\;=\;\omega_{1}^{\prime }\left(0\right)\;=\;\omega_{2}\left(0\right)\;=\;\omega_{2}^{\prime }\left(0\right)\;=\;0,\;\mathrm{and}$$

(7) $$\omega_{1}^{\prime }\left(a_{1}\right)\;=\omega_{2}^{\prime }\left(a_{2}\right)\;=\;0.$$

From Equations (2)–(7), we have:

(8) $$F_{1}=\;\frac{a_{2}^{3}\cdot b_{1}}{a_{2}^{3}\cdot b_{1}+a_{1}^{3}\cdot b_{2}}\cdot \mathrm{ma},$$

(9) $$F_{2}=\;\frac{a_{1}^{3}\cdot b_{2}}{a_{2}^{3}\cdot b_{1}+a_{1}^{3}\cdot b_{2}}\cdot \mathrm{ma},$$

(10) $$\omega_{1}^{\prime \prime }\left(x\right)\;=\;\frac{F_{1}}{4{\mathrm{EI}}_{1}}(a_{1}-2x),\;\mathrm{and}$$

(11) $$\omega_{2}^{\prime \prime }\left(y\right)\;=\;\frac{F_{2}}{4{\mathrm{EI}}_{2}}\left(a_{2}-2y\right).$$

The generated stress along the longtitudinal direction of the sensitive beams and the added beams can be obtained as

(12) $$\sigma_{1}\left(x\right)\;=\;\frac{3a_{2}^{3}\cdot \mathrm{ma}\cdot \left(a_{1}-2x\right)}{2\left(a_{2}^{3}\cdot b_{1}+a_{1}^{3}\cdot b_{2}\right)\cdot h^{2}},\;\mathrm{and}$$

(13) $$\sigma_{2}\left(y\right)\;=\;\frac{3a_{1}^{3}\cdot \mathrm{ma}\cdot \left(a_{2}-2y\right)}{2\left(a_{2}^{3}\cdot b_{1}+a_{1}^{3}\cdot b_{2}\right)\cdot h^{2}}.$$

Then, the maximum stress of the sensitive beams and the added beams are

(14) $$\sigma_{1}(\max)=\sigma_{1}\left(0\right)=\frac{3a_{2}^{3}\cdot \mathrm{ma}\cdot a_{1}}{2\left(a_{2}^{3}\cdot b_{1}+a_{1}^{3}\cdot b_{2}\right)\cdot h^{2}},\;\mathrm{and}$$

(15) $$\sigma_{2}\left(\max\right)\;=\;\sigma_{2}\left(0\right)=\frac{3a_{1}^{3}\cdot \mathrm{ma}\cdot a_{2}}{2\left(a_{2}^{3}\cdot b_{1}+a_{1}^{3}\cdot b_{2}\right)\cdot h^{2}}.$$

The ratio of the sensitive beams’ maximum stress and the added beams’ maximum stress is

(16) $$\frac{\sigma_{1}(\max)}{\sigma_{2}\left(\max\right)}\;=\;\frac{a_{2}^{3}\cdot a_{1}}{a_{1}^{3}\cdot a_{2}}\;=\;\frac{a_{2}^{2}}{a_{1}^{2}}\;=\;{\left(\frac{a_{2}}{a_{1}}\right)}^{2}.$$

Here, the length of the sensitive beams is longer than that of the added beams, so

(17) $$\sigma_{1}(\max)>\sigma_{2}\left(\max\right).$$

That is to say, under the acceleration along the *Z*-axis, the sensitive beams will obtain a larger stress compared with the added beams. Therefore, the stress of the sensitive beams can be investigated as a key parameter that directly determines the accelerometer sensitivity. The maximum strain of the sensitive beams is

(18) $$\varepsilon_{\max}\;=\;\frac{3a_{2}^{3}\cdot \mathrm{ma}\cdot a_{1}}{2E\left(a_{2}^{3}\cdot b_{1}+a_{1}^{3}\cdot b_{2}\right)\cdot h^{2}}.$$

The elastic constant of the structure is

(19) $$K\;=\;2E\left(\frac{b_{1}h^{3}}{a_{1}^{3}}+\frac{b_{2}h^{3}}{a_{2}^{3}}\right).$$

Therefore, the resonance frequency of the accelerometer can be expressed as

(20) $$f\;=\;\frac{1}{2\pi }\sqrt{\frac{K}{m}}\;=\;\frac{1}{2\pi }\sqrt{\frac{2E}{m}\left(\frac{b_{1}h^{3}}{a_{1}^{3}}+\frac{b_{2}h^{3}}{a_{2}^{3}}\right)}.$$

From Equations (18) and (20), it is observed that, if dimensions of the seismic mass and the added beams remain unchanged, the maximum strain of the structure increases with the increase in the length of sensitive beams, while the frequency decreases when the length of the sensitive beams increases. Additionally, as regards the width or thickness of the sensitive beams, the maximum strain and frequency show an opposite result.

To verify the developed model and determine the optimal dimensions of the structure, simulations based on COMSOL software were executed. Then, the location of the piezoelectric thin films was obtained after the optimal dimensions and the placement of the maximum strain were determined.

## 3. Simulation and Analysis

### 3.1. Static Analysis

The dimensions of the piezoelectric accelerometer were determined by measuring an environment that is minute, directional, and low frequency (300~500 Hz). Therefore, the acceleration sensor should satisfy the requirements of high sensitivity (500~600 με), low transverse effect, i.e., a low range (50~100 g), and low resonance frequency (1000 Hz).

**Figure 3:**
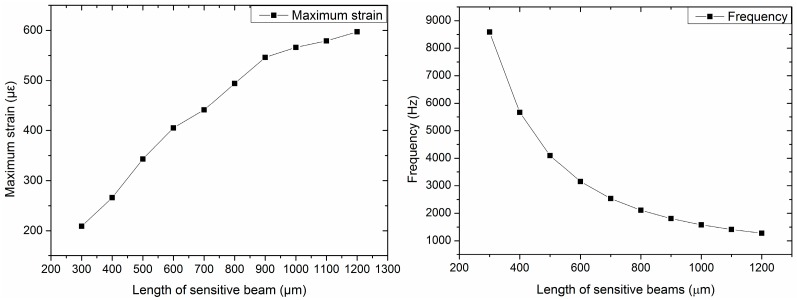
Maximum strain and frequency with different lengths of sensitive beams (thickness is 20 μm; width is 210 μm).

**Figure 4:**
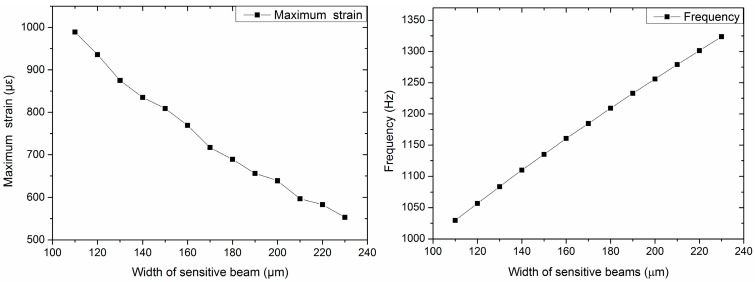
Maximum strain and frequency with different widths of sensitive beams (length is 1200 μm; thickness is 20 μm).

**Figure 5:**
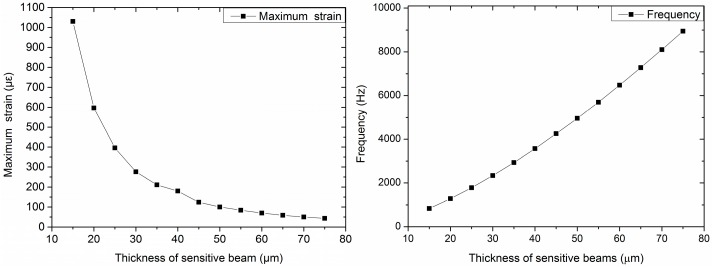
Maximum strain and frequency with different thicknesses of sensitive beams (length is 1200 μm; width is 210 μm).

The designed structure is related to multiple variables. Thus, we used methods of controlling variables to divide the multi-factor problem into single factors. The dimensions of the seismic mass and the length and width of the added beams should be constant. Furthermore, considering the small size of sensors, the dimensions should be as small as possible. In this project, the size of the seismic mass was set to be 2400 μm × 2400 μm × 400 μm. The length of the added beams was 1300 μm, and the width was 100 μm, considering the manufacturing process. Then, the impacts of the length, width, and thickness of the sensitive beams on the sensitivity and frequency are studied as shown in Figure 3, Figure 4 and Figure 5.

Figure 3 shows the maximum strain and frequency of different lengths of sensitive beams when the thickness of the sensitive beams is 20 μm and the width is 210 μm. Figure 4 shows the maximum strain and frequency with different widths of sensitive beams when the length of the sensitive beams is 1200 μm and the thickness is 20 μm. Figure 5 shows the maximum strain and frequency with different thicknesses of sensitive beams when the length of the sensitive beams is 1200 μm and the width is 210 μm. It can be observed that the maximum strain increases as the length of sensitive beams increases. The maximum strain decreases with the increase of the width or the increase of the thickness of the sensitive beams. The results of frequency are opposite compared with the maximum strain, which shows the consistency of the previous theoretical analysis.

To obtain high sensitivity and ensure the linearity and precision of the accelerometer, the maximum strain should not exceed 1/5~1/6 of the strain limit of silicon [13]. That is to say, the strain should be less than 500~600 με. As we can see from the figures, when the size of the sensitive beams is 1200 μm × 210 μm × 20 μm (length × width × thickness), the value of maximum strain is up to 597 με, which is close to 600 με. Therefore, the dimensions of the sensitive beams are 1200 μm × 210 μm × 20 μm.

**Figure 6:**
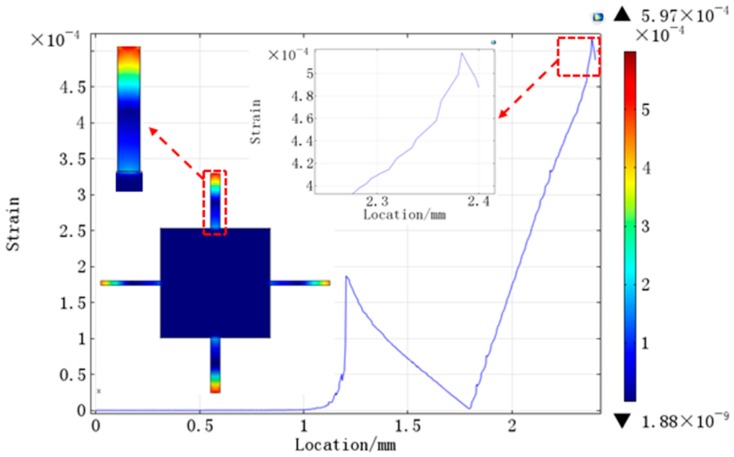
The strain distribution from the center of mass to the edge of sensitive beams.

The strain distribution of the structure is shown in Figure 6. Because there are differences between sensitive beams and added beams in the length and width, the strain distributions of the two kinds of beams are different. It can be observed that the maximum strain is generated on the sensitive beams. In order to get high sensitivity, piezoelectric thin films were distributed at the maximum stress spots. As shown in Figure 6, the value of the strain is from the center of the mass to the edge of the sensitive beams. At the edge of the sensitive beams, the value of the strain is up to maximum and the strain focuses on the area from 2.3 mm to 2.4 mm. In order to get the maximum output voltage, the piezoelectric thin films were arranged in this area.

**Figure 7:**
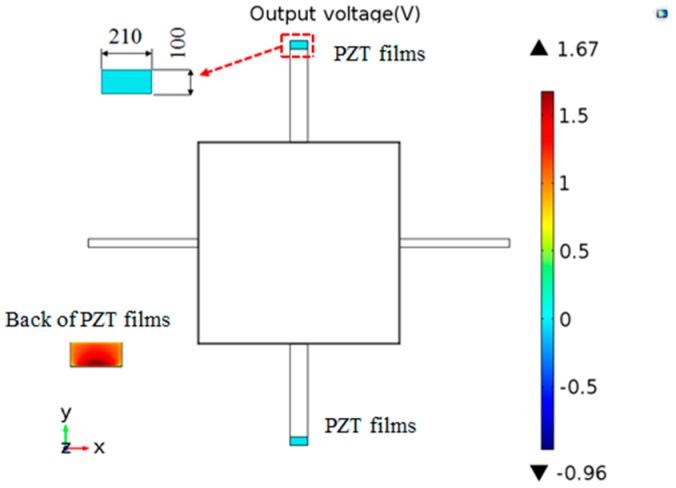
The result of output voltage.

PZT has superior piezoelectric properties compared with other piezoelectric materials [14]. Therefore, this work focuses on the use of PZT films. The width of the PZT films is the same as that of the sensitive beams. The length of the PZT films is 100 μm, which is at the area from 2.3 mm to 2.4 mm. Considering the actual machining precision, the thickness of the PZT films is 3 μm. Moreover, it is supposed that the top surface of the PZT films is zero potential. The output voltage is shown in Figure 7. The maximum output voltage is 1.67 V, which is in the back of the PZT films.

### 3.2. Mode Analysis and Frequency Domain

**Figure 8:**
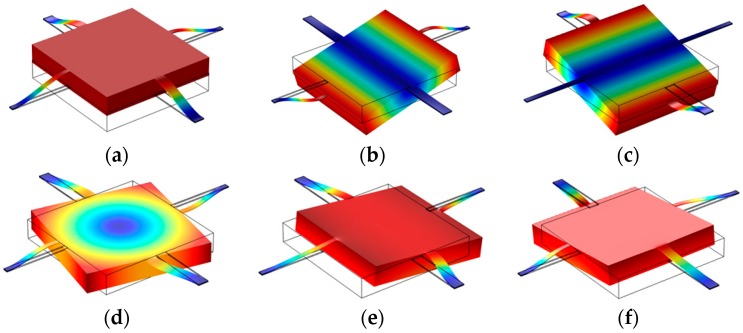
The first to sixth vibration mode of the structure. (**a**) The first mode; (**b**) The second mode; (**c**) The third mode; (**d**) The fourth mode; (**e**) The fifth mode; (**f**) The sixth mode.

Normally, the response of the accelerometer is linear on a wide frequency range [15]. In order to obtain reliable and accurate results, the working frequency of the sensor should be lower than the resonance frequency of the accelerometer. To obtain the resonance frequency and verify which vibration is the dominant one on the accelerometer, mode analysis was carried out, as shown in Table 1 and Figure 8. Table 1 shows that the first natural frequency of the structure was 1279.1 Hz, which is also the working modal of the structure. The first natural frequency is higher than the frequency of ordinary external excitation and distant from other natural frequencies, so the accelerometer could resist the interference of the external signal and obtain the vibration signal accurately.

**Figure 9:**
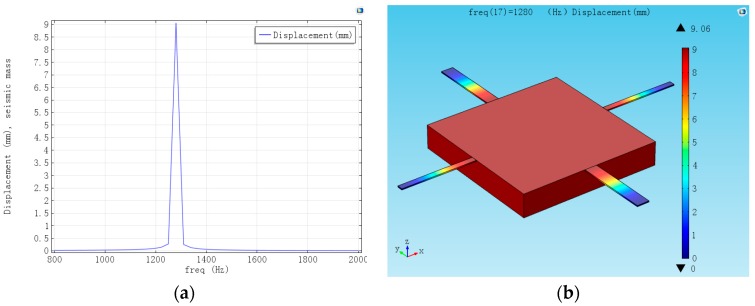
Frequency domain and displacement of the structure in natural frequency. (**a**) Frequency domain; (**b**) Displacement of the structure in natural frequency.

Frequency domain describes the response of the accelerometer at different frequencies. Figure 9 is the frequency domain of the structure. It illustrates that, when the frequency is lower than the natural frequency, with the increase in the frequency, displacement increases. When the frequency equals the natural frequency, the maximum response appears.

### 3.3. Deflection Analysis

Generally, the accelerometer needs to be bonded with a glass to prevent overload. There is a certain space between the seismic mass and the glass to guarantee that the seismic mass can vibrate under normal working conditions. Moreover, it can protect the structure from being destroyed when there is a great impact. Therefore, in order to determine the size of space and the measuring range, deflection analysis was carried out without exceeding the silicon’s stress limit of 450~500 MPa.

**Figure 10:**
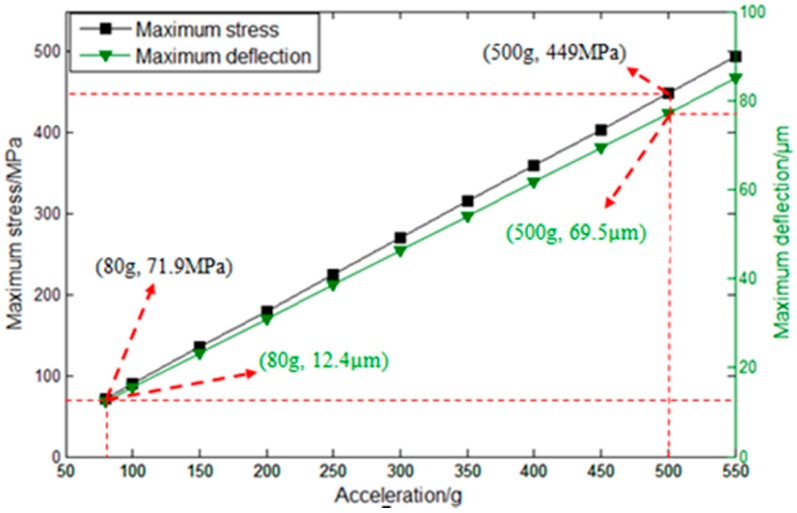
The results of deflection analysis.

The results are shown in Figure 10. It can be observed that the maximum stress and deflection increase with the increase in applied acceleration. When the applied acceleration is 80 g, the value of maximum stress is 71.9 MPa, and the deflection is 12.4 μm. As the acceleration is 500 g, the value of maximum stress is up to 449 MPa, and the deflection is 69.5 μm. In order to ensure that the maximum stress does not exceed the limit stress of silicon, the space between the glass and the seismic mass must be between 12.4 μm and 69.5 μm. The measuring range can be up to 500 g.

### 3.4. Comparisions

We then compared the designed structure with the traditional structures, and the comparisons of the results are summarized in Table 2. The four structures have the same dimensions of the seismic mass and the sensitive beams. The seismic mass’s dimensions are 2400 μm × 2400 μm × 400 μm, and the sensitive beams’ dimensions are 1200 μm × 210 μm × 20 μm. The applied acceleration is 80 g.

It can be observed that, under the same size, the transverse effect coefficients of the structure with a single beam are 0.4932 in the *X* direction and 0.0847 in the *Y* direction. The output voltage is 15 V, which is the highest. However, the maximum strain is 5920 με. It exceeded the strain limit of silicon (3000 με). Thus, under the acceleration of 80 g, the structure was destroyed. In addition, the frequency was too low, which easily generated resonance. Therefore, the bandwidth was narrow and was not suitable for dynamic measurement. The structure with double sensitive beams had low transverse effect coefficient in the *Y* direction (0.1179). However, the transverse effect coefficient in the *X* direction (0.7474) was large. It was easily affected by the acceleration of the *X* direction. In addition, the maximum strain (817 με) exceeded 1/5~1/6 of the strain limit of silicon, which means that the linearity and precision of the accelerometer could not be ensured. The structure with four sensitive beams had low transverse effect coefficient in both *X* and *Y* directions. However, the maximum strain and output voltage were low, and the frequency was relatively higher than other structures.

Compared to other structures, the transverse effect coefficients of the designed structure were 0.0329 in the *X* direction and 0.0992 in the *Y* direction, which were the lowest. This indicates that the designed structure was less affected by transverse acceleration. The value of the maximum strain is 597 με, which is below the strain limit of silicon. The output voltage was 1.67 V, which is relatively high. Moreover, the frequency was also close to 1000 Hz. From comparison of the results, it was found that the structure with two sensitive beams and two added beams not only improves the maximum strain and output voltage but also reduces the transverse effect coefficient.

The structure of the four sensitive beams was completely symmetrical; thus, force applied to the structure split into the four beams. Stress in each beam was small, so the sensitivity was low. However, the sensitive beams of the designed structure obtained a larger stress compared with the added beams. The sensitivity was improved with respect to the structure of the four sensitive beams. Because of the application of the added beams, the stiffness of the structure increased. The transverse effect was reduced compared with the structure of the double sensitive beams. Therefore, the piezoelectric accelerometer has the characteristics of high sensitivity, low transverse effect, and low frequency and can ensure the linearity and precision of the accelerometer.

## 4. Fabrication

**Figure 11:**
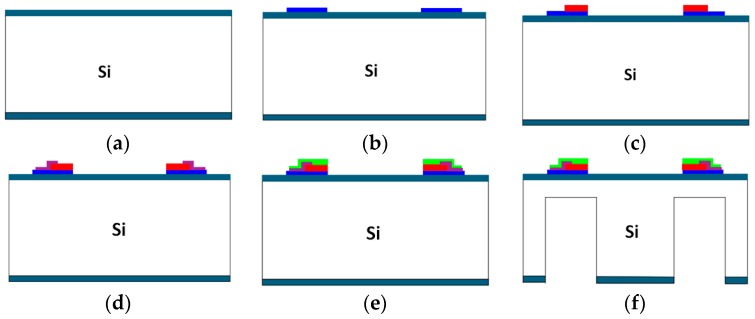
The process of fabrication. (**a**) Oxidated; (**b**) The lower electrodes; (**c**) PZT thin films and RTA; (**d**) Al_2_O_3_; (**e**)The upper electrodes; (**f**) ICP etching.

The piezoelectric accelerometer was fabricated by bulk-micromachining technology, and the fabrication process is shown in Figure 11. First, the (100) oriented n-type Si wafer whose thickness is 400 μm was an oxidated-silicon (SiO_2_) thin film via a thermal oxidization process; Second, the lithography was utilized to expose the glue, and the sputtering method was employed to sputter a layer of chromium/aurum (Cr/Au) as the lower electrode. After that, lift-off was used to form the shape of the lower electrode. Here, Cr was an adhesive layer, which was used to promote the adhesion of Au and Si/SiO_2_; Third, PZT thin films were prepared on the lower electrodes using the lithography, the sputtering method, and lift-off. In the fabrication process, the PZT target was a 3-in-diameter Pb(Zr_0.52_Ti_0.48_)O_3_ ceramic target, and 10% excess of Pb was added to compensate for the losses of Pb during the sputtering; Fourth, the rapid thermal annealing (RTA) was applied to make the PZT thin films from amorphous into crystalline. When the temperature was up to 650 °C at the rate of 50 °C/s, the PZT thin films needed to incubate for 5 min and cool to room temperature; Fifth, a layer of Al_2_O_3_ was deposited at the edge of the PZT thin films, which was to prevent the upper electrodes and the lower electrodes from contacting and causing a short circuit; Sixth, the upper electrodes were made on the PZT thin films using the same methods as the lower electrodes. Seventh, the inductively coupled plasma (ICP) was utilized to etch the front side of the wafer; then, the sensitive beams, added beams, and seismic mass were formed. Finally, the sensitive beams, the added beams, and the seismic mass were released by a back-side ICP process.

**Figure 12:**
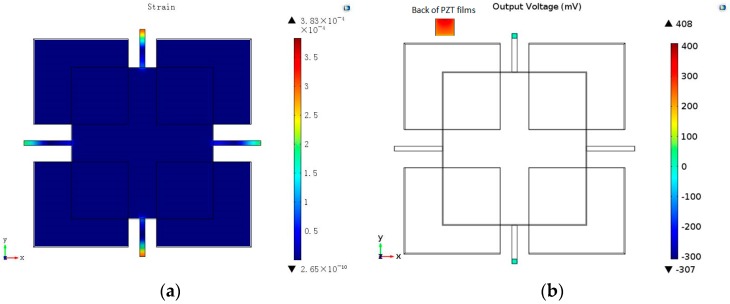
The distribution of maximum strain and the output voltage of the optimal structure. (**a**) The distribution of maximum strain; (**b**) The output voltage of the optimal structure.

**Figure 13:**
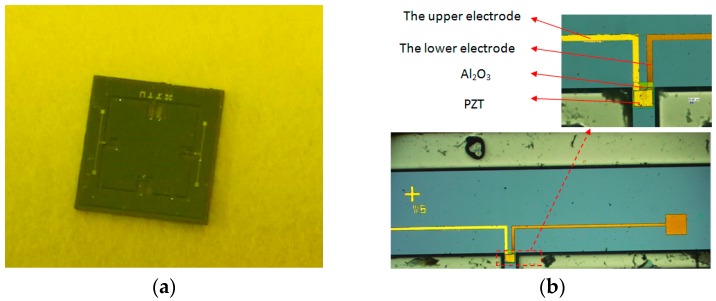
Fabricated piezoelectric accelerometer chip and the enlarged view. (**a**) Fabricated piezoelectric accelerometer chip; (**b**) The enlarged view.

The fabrication was related to six masks. During the process, many chips were fragile. Most of the failures occurred during the release of the sensitive beams, the added beams, and the seismic masses in the final ICP process. The fabrication was expected to be enhanced when thicker suspension beams were used. In order to meet the demands of the fabrication, the thickness of the beams needed to increase from 20 μm to 50 μm, while the maximum strain and the output voltage were down to 100 με and 103 mV, respectively. When the thickness of the beams increased, the sensitivity of the accelerometer decreased as stated above. To improve the sensitivity of the accelerometer, the optimization of the designed structure was conducted. On the basis of the structures of the two sensitive beams and the two added beams, four additional small masses were added to the seismic masses. By increasing the quality of the structure, the sensitivity improved. The distribution of the maximum strain and the output voltage are shown in Figure 12. Maximum strain increased to 383 με, and the maximum output voltage increased to 408 mV. Moreover, the natural frequency was 3313.4 Hz. The fabricated piezoelectric accelerometer chip is shown in Figure 13, and the dimensions of the final structure are described in Table 3.

## 5. Experiment and Results

**Figure 14:**
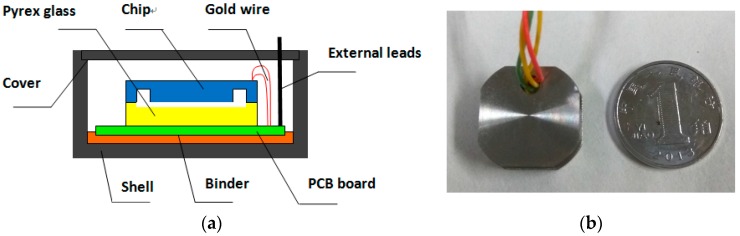
The schematic of the packaging and the photo of the packaged sensor. (**a**) The schematic of the packaging; (**b**) Photo of the packaged sensor.

After the accelerometer chip was fabricated, the key problem was how to realize the package of the sensor. The schematic of the packaging and the packaged accelerometer are shown in Figure 14. First, the chip needed to be bonded with Pyrex glass to prevent overload and then cohered with the PCB. The electrical connection between the pads in the sensor chips and PCB was achieved by gold wire. Finally, the accelerometer chip was completely enclosed in the shell.

**Figure 15:**
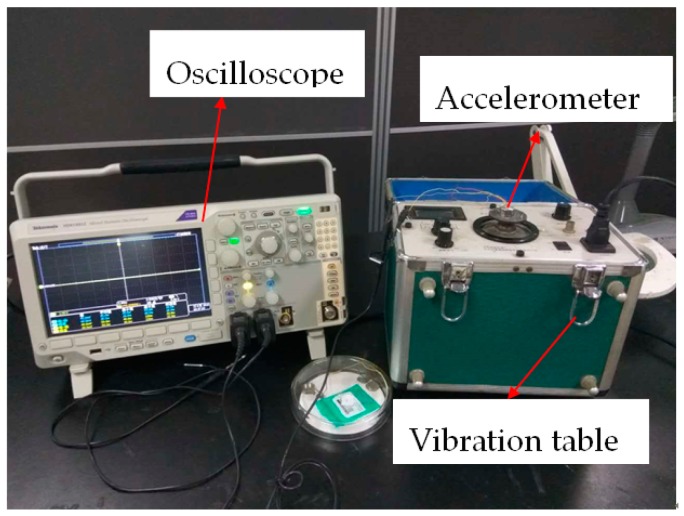
Vibration test.

**Figure 16:**
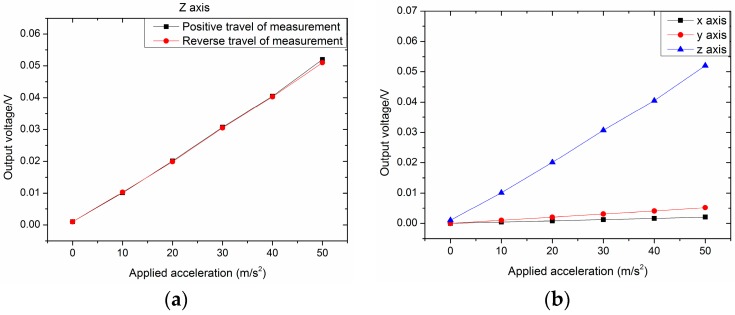
Testing results. (**a**) Positive and reverse travel of measurement; (**b**) Output voltage of the *Z*-, *X*-, and *Y*-axes.

To test the performance of the piezoelectric output of the designed structure, a vibration test was carried out, as shown in Figure 15. The structure was on the vibration table, and different accelerations from 0 m/s^2^ to 50 m/s^2^ at 20 Hz were applied. In order to increase the output voltage, the two piezoelectric thin films were in series. The results are shown in Figure 16a. Obviously, the lines of the positive travel of measurement and the reverse travel of measurement match well; the structure kept the linearity satisfied. Based on Matlab, via calculation, the sensitivity of the piezoelectric acceleration was 0.00091 V/(m/s^2^), the linearity was 0.0205, and the hysteresis error was 0.0033. To measure the transverse motion of the accelerometer, we only needed to change the installation direction of the sensor. The results are shown in Figure 16b. The sensitivity of the *X* direction was 3.91343 $\times \;10^{-5}$ V/(m/s^2^), and the sensitivity of the *Y* direction was 9.78357 $\times \;10^{-5}$ V/(m/s^2^). The results illustrate that the accelerometer is less affected by transverse acceleration.

## 6. Conclusions 

In this paper, a piezoelectric accelerometer was designed, simulated, and analyzed in terms of its maximum stress, natural frequency, and output voltage under an acceleration through the FEM. Moreover, the optimal dimensions were determined. Through the above analysis, it was found that a piezoelectric accelerometer with two sensitive beams and two added beams has the characteristics of high sensitivity, low transverse effect, and low frequency, which meets the requirements of cable fault detection. From mode analysis, the fundamental mode is known and the natural frequency is 1279.1 Hz. In order to obtain reliable and accurate detecting results, the accelerometer must work under the condition that the frequency range is lower than the natural frequency. Through deflection analysis, it was also found that the space between the seismic mass and the glass must be between 12.4 μm and 69.5 μm. Then, the sensor chip was fabricated using lithography, sputtering, ICP technology, and so on. In the fabrication process, we found that most of the chips were fragile during the release of the sensitive beams, the added beams, and the seismic masses in the final ICP process. To improve the yield of fabrication, the thickness of the beams needed to increase. However, the sensitivity decreased. To increase the sensitivity, four additional small masses were added to the seismic masses. Then, the sensor chips were wire-bonged to printed circuit boards (PCBs) and simply packaged for experiments. Finally, the vibration test was carried out, which verifies that the designed structure has good piezoelectric output characteristics. In the future, the designed structure can be optimized to meet different application demands, including the activation of automotive safety systems, machine and vibration monitoring, and biomedical applications for activity monitoring.

## Figures and Tables

**Table 1:** The result of the modal analysis.

Vibration Mode	Frequency (Hz)
The first mode	1279.1
The second mode	1841.5
The third mode	2772.5
The fourth mode	20,421
The fifth mode	45,272
The sixth mode	65,700

**Table 2:** Comparison of the results.

Structures	Transverse Effect Coefficient		Maximum Strain (με)	Output Voltage (V)	Frequency (Hz)
	*X* Direction	*Y* Direction			
Single sensitive beam	0.4932	0.0847	5920	15.00	136.47
Double sensitive beams	0.7474	0.1179	817	2.25	1093.4
Four sensitive beams	0.2053	0.2097	412	1.10	1545.0
Designed structure	0.0329	0.0992	597	1.67	1279.1

**Table 3:** Dimensions of the final structure.

Items	Length × Width × Thickness
The sensitive beams	800 μm × 110 μm × 50 μm
The added beams	1000 μm × 100 μm × 50 μm
Seismic mass	3000 μm × 3200 μm × 400 μm
Four additional small masses	2000 μm × 1800 μm × 400 μm
PZT thin films	100 μm × 110 μm × 1 μm
